# Benefits and costs of oil palm expansion in Central Kalimantan, Indonesia, under different policy scenarios

**DOI:** 10.1007/s10113-015-0815-0

**Published:** 2015-05-28

**Authors:** Elham Sumarga, Lars Hein

**Affiliations:** Environmental Systems Analysis Group, Wageningen University, P.O. Box 47, 6700 AA Wageningen, The Netherlands; School of Life Sciences and Technology, Institut Teknologi Bandung (ITB), Jalan Ganesa 10, Bandung, 40132 Indonesia

**Keywords:** Oil palm expansion, Moratorium, Logistic regression, Ecosystem services, Trade-off, Central Kalimantan

## Abstract

Deforestation and oil palm expansion in Central Kalimantan province are among the highest in Indonesia. This study examines the physical and monetary impacts of oil palm expansion in Central Kalimantan up to 2025 under three policy scenarios. Our modelling approach combines a spatial logistic regression model with a set of rules governing land use change as a function of the policy scenario. Our physical and monetary analyses include palm oil expansion and five other ecosystem services: timber, rattan, paddy rice, carbon sequestration, and orangutan habitat (the last service is analysed in physical units only). In monetary terms, our analysis comprises the contribution of land and ecosystems to economic production, as measured according to the valuation approach of the System of National Accounts. We focus our analysis on government-owned land which covers around 97 % of the province, where the main policy issues are. We show that, in the business-as-usual scenario, the societal costs of carbon emissions and the loss of other ecosystem services far exceed the benefits from increased oil palm production. This is, in particular, related to the conversion of peatlands. We also show that, for Central Kalimantan, the moratorium scenario, which is modelled based on the moratorium currently in place in Indonesia, generates important economic benefits compared to the business-as-usual scenario. In the moratorium scenario, however, there is still conversion of forest to plantation and associated loss of ecosystem services. We developed an alternative, sustainable production scenario based on an ecosystem services approach and show that this policy scenario leads to higher net social benefits including some more space for oil palm expansion.

**Electronic supplementary material:**

The online version of this article (doi:10.1007/s10113-015-0815-0) contains supplementary material, which is available to authorized users.

## Introduction

Oil palm is the most rapidly expanding perennial crop in tropical countries (Phalan et al. 2013). Indonesia and Malaysia supply about 85 % of the world’s palm oil production (RSPO 2013). Oil palm development in Indonesia started in 1911 (Corley and Tinker 2003) with a particularly rapid expansion in recent decades. In the period 2005–2010, oil palm plantations expanded at a rate of 514,000 ha per year and in 2010 the plantations covered 7.7 million ha (Gunarso et al. 2013).

Oil palm development in Indonesia has led to a number of environmental and social concerns (Sheil et al. 2009; Carlson et al. 2012; Hein and van der Meer 2012). At least 56 % of oil palm expansion in Indonesia during the period 1990–2005 has involved the conversion of forests (Koh and Wilcove 2008). Forest conversion, and in particular peat conversion, has led to high carbon (C) emissions, with emissions from land use change making Indonesia the third largest greenhouse gas emitter on the planet (Germer and Sauerborn 2008; Carlson et al. 2013). The conversion of forests has also initiated social problems (Obidzinski et al. 2012). This includes conflicts related to land ownership (Feintrenie et al. 2010) and restricted access to land resources, particularly for local people who traditionally utilise forest products for their daily life. Finally, forest conversion also leads to a loss of biodiversity (Wilcove and Koh 2010). For example, in Indonesia, the population of the orangutan (*Pongo* spp.) has declined substantially, in particular due to habitat loss (Nantha and Tisdell 2009).

There have been various responses aimed at reducing the environmental and social issues associated with palm oil expansion. The Round-Table on Sustainable Palm Oil (RSPO) is an international response supported by a consortium of companies, NGOs, and government agencies, and is resulting in increasing numbers of RSPO-certified plantations and mills that produce palm oil in a more responsible manner. In Indonesia, national instruments include regulations on the maximum peat depth that can be converted to oil palm cultivation (Ministry of Agriculture 2009) and a temporary moratorium on the conversion of peatlands and primary forests (Indonesian President Instruction no. 10 2011; Indonesian President Instruction no. 6 2013). However, at the same time, continued oil palm expansion is promoted by the Ministry of Agriculture which targets an annual expansion rate of oil palm plantation of 2.55 % (Ministry of Agriculture 2011). In view of the high global demand for palm oil products and the profitability of the crop, further expansion of oil palm plantations can be expected in the coming decades (Sheil et al. 2009; Miettinen et al. 2012; Sayer et al. 2012). In Indonesia, the government has also supported the Indonesian Sustainable Palm Oil (ISPO) that was established in 2011. A key factor in the Indonesian policy environment is the current moratorium on the conversion of certain types of forest land (in particular production forests) to other land uses such as oil palm plantations. The moratorium was first applied in 2011 and has been extended in 2013 (each time for 2 years). The discussion on extension of this moratorium is ongoing and has major repercussions for Indonesia’s and even the planet’s greenhouse gas emissions and biodiversity.

This study aims to model oil palm expansion in Central Kalimantan, Indonesia, and analyse its impact on the trade-offs of ecosystem services. In addition to oil palm production, five other ecosystem services are analysed: timber production, rattan production, paddy rice production, C sequestration, and habitat for orangutan, building upon earlier work that we conducted in this area (Sumarga and Hein 2014). Central Kalimantan is selected for several reasons. First, deforestation and oil palm expansion rates in this province are one of the highest in Indonesia (Broich et al. 2011; Ministry of Agriculture 2014). Comparison of land cover between 2000 and 2010 indicates that about 933,000 ha of new oil palms have been established during this period, 474,000 ha of which resulted from converting forests. Second, Central Kalimantan has extensive peatlands (about 3 million ha), with the deepest peat layer reaching 12 m (Wahyunto et al. 2004). Draining peatlands for oil palm plantation significantly contributes to global C emissions. Third, Central Kalimantan provides a habitat to a little over half of the world’s remaining wild orangutan, counting around 33,000 individuals distributed over 15 main populations (Wich et al. 2008). Deforestation in Kalimantan leads to an annual decline of 1.5–2 % in this population (Ministry of Forestry 2007).

We apply a novel modelling approach by integrating inductive and deductive approaches which in most studies are applied separately (Kolb et al. 2013; Widener et al. 2013; Hu et al. 2014; Mas et al. 2014). An inductive approach to land use modelling uses observed trends in land use change to model land use change (Aspinall 2004; Mas et al. 2014). A deductive approach specifies social, economic or policy scenarios or defining rules of behaviour and interaction between agents of land use change (Le et al. 2010; Ralha et al. 2013). We model the effects of three scenarios on ecosystem services supply: (1) a business-as-usual scenario; (2) a moratorium scenario; and (3) a sustainable production scenario, developed on the basis of an ecosystem services approach and two stakeholder workshops conducted in Central Kalimantan. Subsequently, we link land use change to ecosystem services, analysing the trade-off between oil palm expansion and other ecosystem services under different scenarios. Building upon previous work (Sumarga et al. 2015), we analyse the trade-offs in ecosystem services supply in monetary terms, using an ecosystem accounting approach (European Commission et al. 2013; Edens and Hein 2013).

Our study contributes to reaching a better understanding of the environmental, social, and economic impacts of palm oil expansion resulting from land use change. In particular, our study provides a number of new insights in the costs and benefits of different environmental policy options, at the scale of Central Kalimantan province, with potential implications for the overall debate on the moratorium policy in Indonesia. Our work is also relevant for the sustainability discussions conducted in the context of the RSPO and ISPO, where impacts resulting from land use change have been proven relatively difficult to tackle (Sheil et al. 2009).

## Methods

### Study area

Central Kalimantan is the third largest province in Indonesia covering 15,356,400 ha. The province has a moist tropical climate and is located at latitude 0^o^45′ North–3^o^30′ South and longitude 110^o^45′–115^o^50′ East. The province has a total population of 2,1459,000 with an employment rate of 68 % in 2013. GDP per capita is about €1940 (Statistics Indonesia 2014), and agriculture is the main economic sector, with rice, oil palm, and rubber as the main crops. In addition, mining (coal, gold) and tourism are increasingly important. A land cover map of Central Kalimantan is presented in Appendix 1.

### Spatial modelling of oil palm expansion and its impacts on ecosystem services

This study integrates three modelling parts: regression of the spatial pattern of oil palm expansion, land use scenarios, and impacts of oil palm expansion on the trade-offs of ecosystem services (see Appendix 2 for the modelling framework).

### Spatial pattern of oil palm expansion

Considering the binary response variable (the presence and absence of oil palm expansion), we applied a logistic regression to model spatial patterns of oil palm expansion in the past (2005–2010) and used the model to predict oil palm expansion in 2015, 2020, and 2025. The general model for the logistic regression is given by Formula 1.

1 $$p = \frac{1}{{1 + e^{{ - \left( {\beta_{0} + \beta_{1} x_{1} + \beta_{2} x_{2} \cdots + \beta_{\text{i}} x_{\text{i}} } \right)}} }}$$

where *p* = presence probability (indicating conversion to oil palm) *β*_0_, *β*_1, …_*β*_i_ = coefficients *x*_1_, *x*_2_, … *x*_i_ = values of predictors.

We first overlaid the land cover map of 2005 with the map of 2010 to identify the oil palm expansion areas during that period. The land cover map of 2010 is the most recent land cover map that contains detailed information on oil palm distribution. Next, we randomly generated 1000 presence points within the expansion areas, and 1000 absence points outside the existing oil palm and the expansion areas. Random sampling is an unbiased sampling technique that ensures that each point has the same probability of being selected (see also Aspinall 2004 and van Gils et al. 2008 for random point generation). We related oil palm expansion to six predictors: elevation, soil types, distance to the existing oil palm areas (and thereby to oil palm mills), distance to roads, distance to rivers, and distance to settlements. We recognised the potential change in roads and settlements during 2010–2020, however, considering the difficulty of projecting new roads and settlements, we treated roads and settlements as static. All maps of the predictors are in raster format with a spatial resolution of 100 by 100 m. The predictors were selected to represent the physical factors that are often considered in the selection of new locations for oil palm plantations. We extracted the values of the six predictors in the selected presence and absence points, and ran the logistic regression. The logistic regression was run using the GLM method “R” with the binomial link family (Hastie and Pregibon 1992). The model accuracy was approached by measuring the sensitivity, specificity, and Area Under ROC (Receiver Operating Characteristic) Curve (AUC), see Appendix 3.

### Land use scenarios

We developed three land use scenarios for the use of government land. Government land covers 97 % of Central Kalimantan, and it is here where the main policy issues are. All forest land is government-owned in Central Kalimantan (as in Indonesia as a whole), and the government also owns the large majority of the peatlands (Central Kalimantan Forestry Service 2013). Hence our study does not cover the conversion of privately owned land, including agricultural land owned by smallholders, to oil palm. Since this type of land conversion in most cases does not lead to deforestation or drainage of peat, and since it does not lead to a loss of access of land to local stakeholders, the social costs of this kind of land use change are usually small (and the economic benefits may be substantial, in particular if unproductive land is converted to oil palm; e.g. Colchester et al. 2006). Note that, in Central Kalimantan (as well as in other parts of Indonesia), there are widespread agricultural settlements and encroachments on land that is officially classified as forests. These lands are included in our analysis, as they are government-owned.

Our first scenario is a **business-as-usual scenario (BAU)**. It is assumed that the moratorium is lifted per mid-2015, and oil palm expansion is allowed in all areas currently proposed by oil palm companies and located in “*Areal Penggunaan Lain*” (APL: ‘other forest land’), including in primary forests and peatlands. APL is state land allocated to any use other than conservation forests, protected forests, and production forests. Note that APL land may include good-quality secondary or primary forests. The conversion of land classified as “production forests” is not allowed in this scenario, in line with government regulations (Indonesian Republic Law no. 41 1999).

In the second scenario, the **moratorium scenario (M)**, we assume that the current forest conversion moratorium is extended to 2025. In this scenario, oil palm expansion in primary forests and peatlands is prohibited. The boundaries of primary forest are indicated in the 2010 land cover map of the Ministry of Forestry (2011, unpublished) and the boundaries of the peatlands are from Wahyunto et al. (2004). Note that, in line with current practices, land that can be converted under the moratorium includes good-quality secondary forest, as long as it is classified as APL land. Production forests cannot be converted in this scenario.

The third scenario is an alternative **‘sustainable production’ scenario (SP)**. This scenario was developed using an ecosystem services approach, building on inputs received during two stakeholder workshops conducted one each in two districts (West Kotawaringin and Kapuas) in February and March 2014. These stakeholder workshops were attended by 48 participants from both government agencies (Forestry, Agriculture, Environment, Planning, Development Economic, National Park) and non-government organisations (Orangutan Foundation International, Friends of National Parks Foundation, local community, journalist). These stakeholders indicated that the moratorium was not sufficient to arrest forest degradation and that at the same time there is a need to better regulate oil palm expansion. They proposed to examine an approach where ecosystem services would be integrated in the policy framework. In this scenario, both production and APL forest can be converted to oil palm plantations provided that: no primary forest or peatland is converted and no key ecosystem services are lost. The latter has been interpreted by the researchers as prohibiting the conversion of (1) land used for timber production; (2) rattan fields; (3) croplands (illegally) established in forest land; (4) forest land with a C content at least as high as in a mature palm oil plantation (i.e. 51 ton C/ha); and (5) orangutan habitat (as mapped in Sumarga and Hein 2014).

### Impact of oil palm expansion on ecosystem services supply

The implications of the three scenarios for ecosystem services supply were analysed for the period 2015–2025. Given that there is no map indicating oil palm expansion in Central Kalimantan that is more recent than 2010, we mapped oil palm expansion during the period 2010–2015 using the logistic regression model under the moratorium policy that has been in place in the past years. We used the raster calculator of ArcMap 10.1 to apply the logistic regression model with the six layers of predictors as inputs. For the distance to existing oil palms predictor, we used oil palm distribution in 2010 as a reference. We overlaid this map with the map of land availability for oil palm expansion from the M scenario, resulting in the predicted areas of oil palm expansion during 2010–2015. We combined this map with the oil palm map of 2010 to generate the oil palm extension map of 2015, which was then used as a reference for predicting oil palm expansion in the period 2015–2020. To model the expansion up to 2020, we created the map with the distance to oil palm in 2015 and used it as one of the six predictors. We then overlaid the predicted expansion from the logistic regression model with the land availability from the three scenarios to generate the three corresponding maps indicating oil palm expansion up to 2020. We applied the same procedures to analyse oil palm expansion in the period 2020–2025 and map oil palm extent in 2025, for the three scenarios.

The trade-offs of ecosystem services were analysed in terms of physical quantities and monetary values. We used the physical quantities and the monetary values derived from maps of ecosystem services prepared by two previous studies (Sumarga and Hein 2014; Sumarga et al. 2015; see Table 1). Our monetary analysis uses the valuation approach of the national accounts (European Commission et al. 2009), in line with these previous studies. This valuation approach is based on exchange values and excludes consumer surplus (Edens and Hein 2013; European Commission et al. 2013). We come back to the implications of our valuation approach in the Discussion section. As indicator for monetary value of timber, palm oil, rattan, and rice, we used the resource rent generated by the crop, reflecting the contribution of the ecosystem to the production of this crop, expressed on a per ha basis. The resource rent requires subtracting the costs of intermediate inputs and labour, and the user costs of fixed capital, from the gross farm-gate revenues (see e.g. Edens and Hein 2013). For the costs of C sequestration, we used the social costs of C from the US EPA (Interagency Working Group on Social Cost of Carbon, United States Government 2013), with an exchange rate of $ 1.33 for € 1 (average in 2010). Orangutan habitat was not expressed in a monetary value in view of the difficulties in assigning a monetary value to biodiversity habitat (e.g. Sumarga et al. 2015). Note that the widely applied contingent valuation method (Loureiro and Ojea 2008; Jacobsen et al. 2012) is incompatible with ecosystem accounting principles. Note that costs of land (and land concessions) are not included in the resource rent and the monetary assessment is net of taxes and subsidies. Hence, our valuation study provides an analysis of costs and benefits at the level of society, which includes state, companies, and smallholders. Net benefits of land use options for individual companies or smallholders may be lower (due to the costs of obtaining land, or taxes) or higher (in case of subsidies).

**Table 1:** Provincial averages of values for ecosystem services, values are presented in terms of physical quantities and monetary values

Ecosystem services	Provincial average	
	Physical quantity	Monetary value
Newly planted oil palm (0–4 years)	3.6 ton/ha/year^c^	Resource rent of € −646/ha/year (on mineral soil) and € −924/ha/year (on peat soil) reflecting costs for establishing the plantations^c^
FFB production of young oil palm (0–9 years)	15.2 ton/ha/year^c^	Resource rent of € 761/ha/year (on mineral soil) and € 509/ha/year (on peat soil)^c^
FFB production of mature oil palm (0–20 years)	24 ton/ha/year^c^	Resource rent of € 1770/ha/year (on mineral soil) and € 1571/ha/year (on peat soil)^c^
Timber production	0.86 m^3^/ha/year^a^	Resource rent of € 35/m^3b^
Rattan production	0.79 ton/ha/year^a,e^	Resource rent of €104/ton^b^
Paddy rice production	2.2 ton/ha/year^a^	Resource rent of € 130/ton^b^
Carbon sequestration	Detailed information in Appendix 1	a Social cost of C of € 88/ton C^b^
Orangutan habitat	Habitat suitability map of orangutan^a^	Not assessed^d^

^a^Sumarga and Hein (2014); ^b^ Sumarga et al. (2015); ^c^ Sumarga et al. (2015) with an assumed increase in productivity of 20 %, the negative resource rent reflect the costs of establishing the oil palm plantations including costs for land preparation, planting and plantation maintenance; ^d^ not assessed due to methodological difficulties, see explanation in the text; ^e^ rattans, dominated by *Calamus manan* and *Calamus caesius*, are planted in secondary forest with a typical maximum distance of 25 km from settlements and 4 km from rivers

We assumed a constant productivity for timber, rattan and paddy rice in all years of the analysis. For oil palm, we assumed that new varieties of oil palm with a higher productivity will gradually be introduced, reaching an average productivity of 24 ton fresh fruit brunch (FFB)/ha/year (Indonesian Oil Palm Research Institute 2015) in mature oil plants for newly planted oil palms in 2025. This productivity is about 20 % higher than the current average productivity. Finally, we assume constant prices for crop inputs (labour, equipment, intermediate inputs) and crop prices. This, of course, is a major simplification that causes some uncertainty in our results. However, meaningful forecasts of price changes in these factors are not available.

## Results

### Logistic regression model

The coefficients of the logistic regression model are as follows: intercept (2.76), elevation (−1.685e-02), distance to roads (−9.477e-06), distance to rivers (1.048e-04), distance to settlements (−6.139e-05), distance to existing oil palm (−3.572e-05), and distance peat soil (−6.432e-01), see Appendix 3 for detailed information. With the full model (all variables are included), the coefficients indicate that the areas with a low elevation, close to roads, close to settlements, close to existing oil palm plantations, and close to mineral soil are preferred for the expansion. The model provides a high spatial accuracy with a sensitivity of 0.89, a specificity of 0.79, and an AUC of 0.9 (see Appendix 3 for the explanations).

### Areas planted with oil palm in 2025

**Fig. 1:**
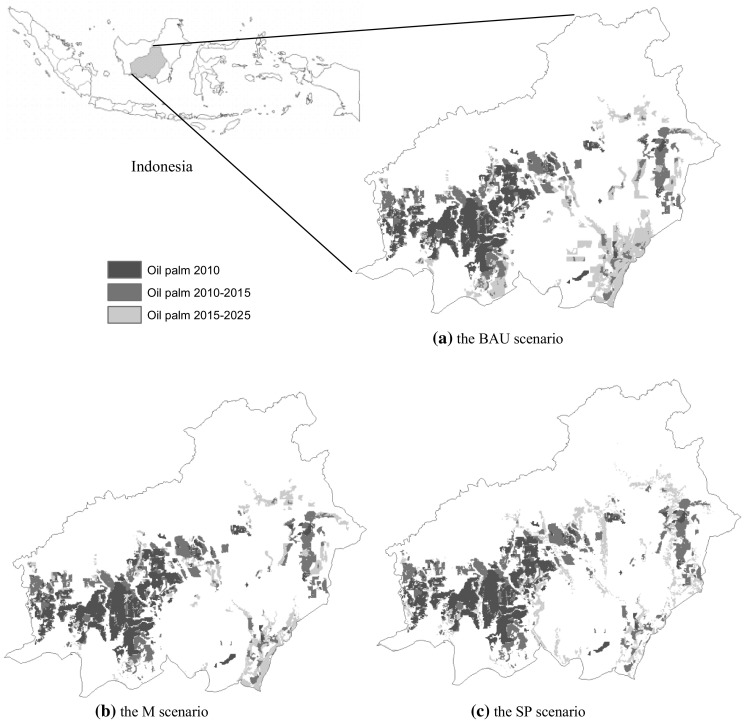
Oil palm expansion according to three scenarios: the BAU, M, SP scenarios (see "Land use scenarios"section for descriptions of the scenarios)

Maps of oil palm expansion up to 2025 are presented in Fig. 1. In the BAU scenario, the predicted new oil palm area during the period 2015–2025 is 1,233,900 ha. In the SP scenario, about 698,700 ha of new oil palm areas will be planted in that period. The M scenario provides the lowest estimate, i.e. 637,800 ha newly planted oil palm. The M scenario gives a lower estimate of expansion areas than the SP scenario since state land with status as “Production forest”, even if it is degraded, cannot be converted in the M scenario, but this type of land can be converted in the SP scenario.

There has been an exponential growth of oil palm areas in Central Kalimantan from 257,000 ha in 2000 to 394,000 ha in 2005 and 1,200,000 ha in 2010. Our model forecasts strong oil palm expansion continuing in the period 2015–2020, in particular in case of the BAU scenario. This growth would, in all three scenarios, level off in the period 2020–2025 (see Appendix 4). The main reason for this is that only limited land remains available for oil palm expansion in this period, depending upon the regulations (and enforcement of these regulations) that drive land use change.

### Impact of oil palm expansion on ecosystem services supply

Trade-offs in ecosystem services supply from oil palm expansion are influenced by three main factors: the rate of land use change, the land cover of the converted areas, and the soil types of the converted areas (mineral or peat). Acreage converted, and land cover and soil types of the converted areas determine the types, quantities, and values of ecosystem services that will be lost due to land use change. The C balance is influenced, in particular, by the amount of forests and peatlands converted to oil palm (see Appendix 5). The results of our analysis, for the three scenarios, are summarised in Table 2, with detailed calculations presented in Appendix 6a–6c.

**Table 2:** Trade-offs in ecosystem services supply from oil palm expansion in different scenarios for the period 2015–2025, negative values indicate depletions of ecosystem services in terms of both physical quantities and monetary values; values are rounded

Ecosystem services	BAU scenario		Moratorium scenario		SP scenario	
	Physical quantity	Monetary value	Physical quantity	Monetary value	Physical quantity	Monetary value
FFB production of oil palm	17.3 Mton/year	€ 627.4 million/year	8.8 Mton/year	€ 377.5 million/year	10 Mton/year	€ 463.7 million/year
Timber production	−0.31 mega m^3^/year	−€ 10.9 million/year	−0.18 mega m^3^/year	−€ 6.3 million/year	No change	No change
Rattan production	−0.34 Mton/year	−€ 35.2 million/year	−0.3 Mton/year	−€ 32.0 million/year	No change	No change
Paddy rice production	−0.49 Mton/year	−€ 63.8 million/year	−0.27 Mton/year	−€ 35.2 million/year	No change	No change
Carbon sequestration	−16.7 Mton C/year	−€ 1466 million/year	−0.9 Mton C/year	−€ 80.0 million/year	1 Mton C/year	€ 92.2 million/year
Orangutan habitat	−102,000 ha		−10,000 ha		No change	

Mton indicates million metric ton

In the BAU scenario, 450,000 ha of forests, 428,000 ha of rattan field (mostly in forests), and 223,000 ha of paddy rice areas are estimated to be converted to new oil palm plantations in the period 2015–2025. The model forecasts that oil palm expansion will take place in about 541,000 ha of peatlands, covering 215,000 ha of forests and 326,000 ha of non-forest areas, and replace about 102,000 ha of orangutan habitat. This can be classified as a substantial impact, considering that this would lead to a loss of close to 10 % of the current habitat in this province and that Central Kalimantan contains over half of the global orangutan population. The effects on ecosystem services supply are summarised in Table 2. This scenario provides the highest increase in monetary value from oil palm production, but on the other hand leads to the highest social costs, in particular, from C emission (and orangutan habitat). Overall, in this scenario, the societal costs (an aggregate value of € 1.5 billion/year from carbon emissions and the loss of production of timber, rattan, and paddy rice) far exceed the societal benefits (a value of € 627 million/year from the increase of oil palm production).

In the M scenario, 212,000 ha of forests, 390,000 ha of rattan field, and 123,000 ha of paddy rice areas will be converted to oil palm plantations. The expansion will replace about 10,000 ha of orangutan habitat. This scenario leads to the lowest increase in oil palm production. Since oil palm expansion on peatlands is not allowed in this scenario, the net C emissions from this scenario are much lower than the emissions in the BAU scenario. This scenario has an overall net societal benefit; the benefits from expansion of palm oil production (€ 377 million/year) exceed the costs related to a loss of other ecosystem services (€ 153 million/year).

In the SP scenario, oil palm expansion will only take place in degraded lands with mineral soils. Hence there will be no change in forested areas, rattan field, paddy rice areas, and orangutan habitat. This scenario provides a positive net C balance, because C storage in oil palm plantations exceeds C storage in degraded lands and no peatland is allowed to be converted. Compared to other scenarios, the SP scenario provides the highest net monetary value, which is € 556 million with no degradation of the areas currently suitable for orangutan habitat.

## Discussion

### Modelling approach

There are a wide range of approaches to model land use change (Overmars et al. 2007; Pontius et al. 2008; Gutzler et al. 2015). Selection of the appropriate modelling approach needs to be based on the specific research objectives, the physical characteristics of the landscape and the ecosystem services it supplies, the scale of the analysis, the key drivers for land use change and (spatial) data availability. Our approach integrates deductive and inductive modelling elements and integrates both spatial predictors and policy scenarios in the model. In the case of oil palm expansion in Indonesia, both aspects need to be considered because the expansion is driven by a combination of physical, social, and political factors (McCarthy and Cramb 2009).

Our model examines only the conversion of land to oil palm (contrary to more generally applicable land use models such as for instance CLUE-S, see Verburg and Overmars 2007). Instead of analysing demand for different land uses, and the relative suitability of each pixel to fulfil that demand, our model only predicts the probability of an area (pixel) to be converted into oil palm, with time steps of 5 years. For modelling a specific type of land use conversion (in our case conversion to oil palm), we believe our model is a suitable alternative to other modelling approaches, because it allows using both scenarios and a comprehensive set of variables to predict land use change and because the significance of each variable can be retrieved. In addition, our model does not require the prior definition of land demand (i.e. the demand for specific types of land use) as a driver for land use change. In the case of oil palm expansion, such land demand would be very difficult to estimate, given the global market for oil palm and the wide range of potential areas that can be used for oil palm across the globe.

In spite of the wide attention that land use change from oil palm expansion received, there are few other studies with which we can compare our results. Carlson et al. (2013) provide estimates of oil palm expansion in the whole of Kalimantan in 2020 based on three scenarios: a BAU scenario, a peatland protection scenario, and a forest protection scenario. By using the extent of oil palm plantations in 2010 as a baseline, they estimated that oil palm areas in 2020 will increase with about a factor 3.7 in the BAU scenario, a factor 3.1 in the peatland protection scenario, and a factor 1.9 in the forest protection scenario. Their BAU forecast is somewhat higher than the estimate of the BAU scenario in our study (in our BAU scenario, the area covered by oil palm in 2020 is about a factor 2.9 higher than in 2010). Our other scenarios (M, SP) result in an expansion of oil palm plantations (between 2010 and 2020) within the range specified by the two environmental protection scenarios of Carlson et al. (2013): an increase with a factor of 2.4 and 2.5 for, respectively, the M and the SP scenarios. Note that the difference between the amount of ha converted in both the M and SP scenario is small, but there is a difference in the specific areas that are converted (see Fig. 1).

### Scenario comparison and policy implications

Oil palm development brings both significant societal benefits and costs. For example, oil palm production can be a major driver for local and national economic development and the crop generates substantial export value (Koh and Wilcove 2007; Obidzinski et al. 2012). It can also increase local employment opportunities and allow local smallholder farmers to benefit from improved infrastructure (such as the presence of oil palm mills) (Sandker et al. 2007). On the other hand, the rapid and often uncontrolled expansion of oil palm plantations in Indonesia is causing costs to society related to a loss of access to land for local people, pressure on infrastructure (in particular from trucks transporting CPO) and environmental impacts. This study only examines societal costs of the environmental impacts related to land use change, including C emissions, loss of timber and non-timber forest production, loss of cropland, and biodiversity habitat loss.

A main challenge for Indonesian policy makers and government officials is to facilitate the expansion of oil palm while minimising the social and environmental costs, as also discussed in the context of the RSPO and ISPO. The RSPO criteria are relatively effective in enhancing social and environmental management in plantations (e.g. regulate pesticide use), and also include several simple criteria to discourage negative effects from land use change, in particular a ban on establishing plantations in primary forest. However, from a land management perspective, the criteria are not yet adequate, since (1) very little primary forests remain, and the conversion of good-quality secondary forests also brings significant environmental costs such as biodiversity loss, habitat destruction, and C emissions; (2) the conversion of shallow or deep peat is not restricted by the RSPO criteria; and (iii) the effects of land use change are determined by the aggregate effect of individual land conversions (and the spatial pattern of such conversions) and are therefore difficult to assess or mitigate at the level of individual plantations. Hence, government intervention remains essential for an effective regulation of palm oil expansion. Moving towards better regulation requires a significant effort from the side of the Indonesian government including enhanced land use planning, continuous development and improvement of regulations for land management and land conversion, monitoring of land use change, and enforcement of the regulations (Wicke et al. 2011; Smit et al. 2013; Lee et al. 2014).

Our scenario analysis may provide useful information for land use planning, since it compares the potential societal benefits (from palm oil expansion) and the societal costs (due to selected environmental impacts) in different policy scenarios. We show that, in the case of Central Kalimantan, the moratorium has important economic benefits for society at large. The benefits of the moratorium, in particular from reduced CO_2_ emissions, far outweigh costs of foregone oil palm expansion. We also show that the most important environmental issue with regard to land conversion to oil palm plantations is the conversion of peat, where the impacts of CO_2_ emissions are largest and also many other ecosystem services including biodiversity habitat are located. Note, however, that our comparison is incomplete: we do not assess social costs (loss of access to land, social changes in society, etc.) and benefits (local employment opportunities) and associated economic costs (impacts on infrastructure from CPO trucking) and benefits (multiplier effects resulting from local economic development). In addition, we only analyse selected ecosystem services. Our analysis does not include for instance the growth of other (agroforestry) crops on mineral or peat soil (e.g. jelutung, *Dyera costulata*), tourism and recreation, fisheries and aquaculture (which is an important economic activity in Central Kalimantan including in lakes and rivers in peatlands, see van Beukering et al. (2008) and Suyanto et al. (2009)), biodiversity other than orangutan habitat, or the impacts of oil palm plantations or mills on water pollution. We also do not consider price changes in ecosystem services. A continuing rapid expansion of oil palm, at the rates experienced in the past years in Indonesia, as well as increasing production in other countries such as Colombia could lead to lower prices for palm oil in the future, whereas prices of other ecosystem products such as timber and rattan may increase over time due to increasing scarcity. In this case, the relative benefits from other services, and the societal costs in the BAU scenario, would be underestimated in our study.

In addition, our study does not consider the effects of the drainage of peatlands. Drainage (of at least 80–90 cm) is required to grow oil palm in peat soils which leads to soil subsidence in the order of 3–5 cm per year (substantially more in the first year following drainage; Wösten et al. 2008; Hooijer et al. 2012). Over time, this will affect water flows and flood risks, and it may well render the peatland unsuitable for oil palm in the course of one to several decades because rain or river water accumulates in the drained peatlands which have become the lowest-lying areas in the landscapes. This omission also means that we are very likely to overestimate the net benefits of oil palm plantations and to underestimate the benefits from other land uses, in particular in the peatlands.

We compared trade-offs in ecosystem services supply between the three policy scenarios (Table 2) using an accounting approach to value ecosystem services (European Commission et al. 2013). In particular, this approach measures the value of the contributions of ecosystems to economic activity (including consumption and production) in an approach that is applicable at aggregated scales such as the whole of Central Kalimantan province (Edens and Hein 2013; Obst and Vardon 2014). However, since this approach excludes consumer surplus, we underestimate the total economic value generated by ecosystems in the province, such as the value accruing to consumers of palm oil or rice because of a lower price compared to a situation with less oil palm production in Central Kalimantan (which provides 11 % of the national oil palm production and 1.2 % of the national rice production). Our study indicates that the costs of C emissions alone substantially exceed the benefits of oil palm expansion in the BAU scenario. The other ecosystem services (and the aspects not monetised such as habitat loss and other ecosystem services that we did not consider in our study) point to this scenario being the least preferred from the perspective of society at large. A critical point is, however, that the costs of C emission are not directly paid by the emitter. Costs are born over the longer term, by all countries that will face the impacts of climate change (including Indonesia which has a high population density in its low-lying coastal zones). This, again, points to the need to establish markets for C, as well as to the need to prioritise the protection of peatlands from drainage (Agrawal et al. 2011; Hooijer et al. 2012).

The moratorium, in our analysis, is not able to arrest the conversion of well-preserved secondary forest to other land uses such as oil palm. This explains the CO_2_ emissions that still take place in the M scenario, which includes the conversion of 212,000 ha of forest land to oil palm. The converted forest in this scenario has a C storage more than 100 ton C/ha and represents well-preserved secondary forest with potentially the capacity to return to full forest cover. Still, the M scenario is a lot better than the BAU scenario with a positive economic impact for society at large. From a social planner perspective, SP is the preferred scenario. This scenario leads to minimal impacts on ecosystem services, an important expansion of oil palm, and a net increase in C storage. An important consideration, however, is that enforcement of policies is critical and that further work is needed to examine how an SP scenario could be enforced given that there are few maps of ecosystem services supply in the province, and that enforcement has often proven to be complex in the Indonesian policy context (Sayer et al. 2012). It is also important to consider that such a policy scenario may lead to the perverse incentive of degrading forest so as to reduce ecosystem services supply and facilitate obtaining a license for land use conversion.

## Conclusion

We modelled oil palm expansion in Central Kalimantan and analysed its effects on the supply of ecosystem services in three scenarios: a business-as-usual, moratorium, and sustainable production. We analysed the effects of land use change on six ecosystem services: oil palm, timber, rattan, paddy rice, C sequestration, and orangutan habitat, and we analysed these effects in both physical quantities and monetary values. We modelled land use change based on an integrated inductive and deductive approach that combines a spatial logistic regression model with a set of rules governing land use change as a function of the policy scenario. Our study shows that in all scenarios there will be a continued rapid increase in oil palm production in Central Kalimantan. In the case of the BAU scenario, however, this expansion would lead to substantial net costs to society resulting from a loss of ecosystem services and in particular C emissions. Continuation of the moratorium leads to a positive net benefit for society. However, there is still a conversion of forest, even with this moratorium in place. A sustainable production scenario, which was developed using inputs from two stakeholder workshops, provides for an alternative—although difficult to enforce—policy scenario. In this scenario, land use change is restricted to areas where impacts on ecosystem services would be minimal, and this would have the highest net societal benefits.

## Electronic supplementary material

Below is the link to the electronic supplementary material.

Supplementary material 1 (DOC 530 kb)
