# Supplementary material for: Benefits and costs of oil palm expansion in Central Kalimantan, Indonesia, under different policy scenarios
Source: Reg Environ Change. 2015 May 28;16:1011–21. doi: 10.1007/s10113-015-0815-0 (PMC4927089; doi:10.1007/s10113-015-0815-0)
Supplement: Supplementary file 1 — Supplementary material 1 (DOC 530 kb) [file 10113_2015_815_MOESM1_ESM.doc]

Appendix 1. Location of Central Kalimantan and its dominant land cover types derived from land cover map 2010 (Tropenbos Indonesia 2011, unpublished)


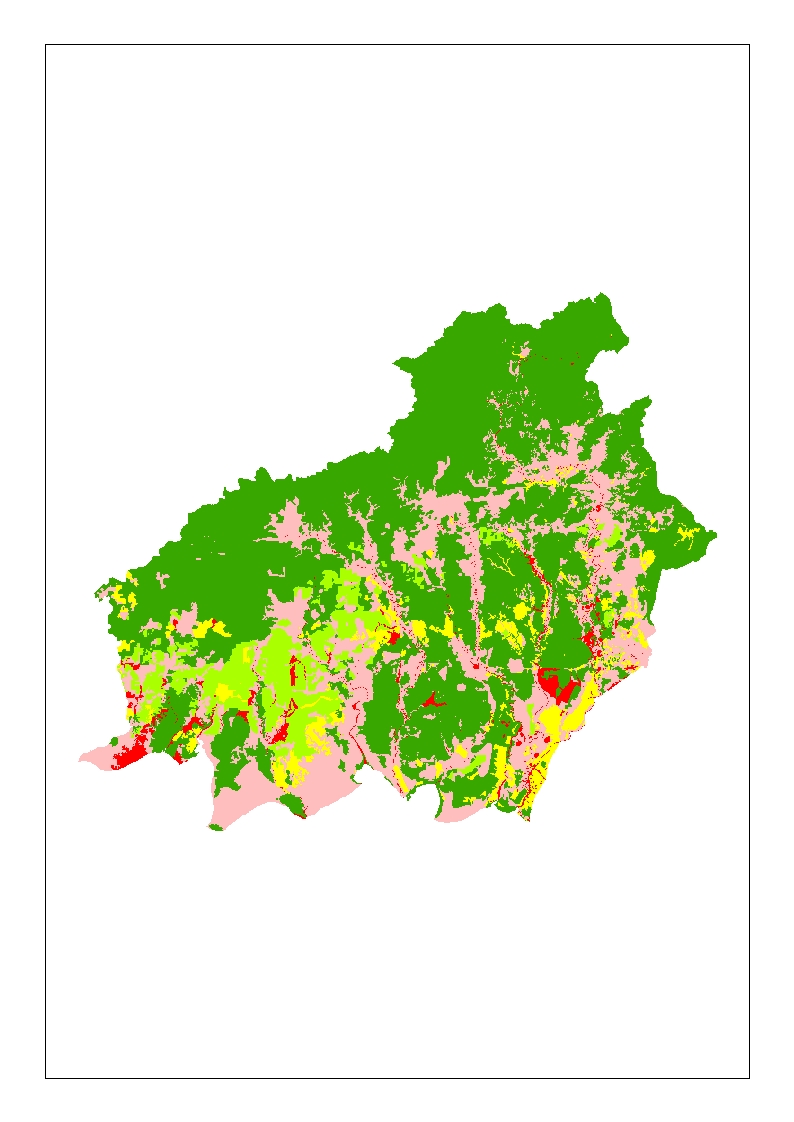

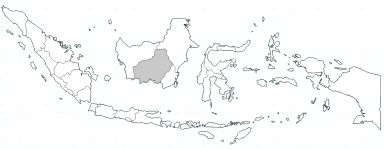


Appendix 2. Framework for modelling oil palm expansion and its impact to ecosystem services


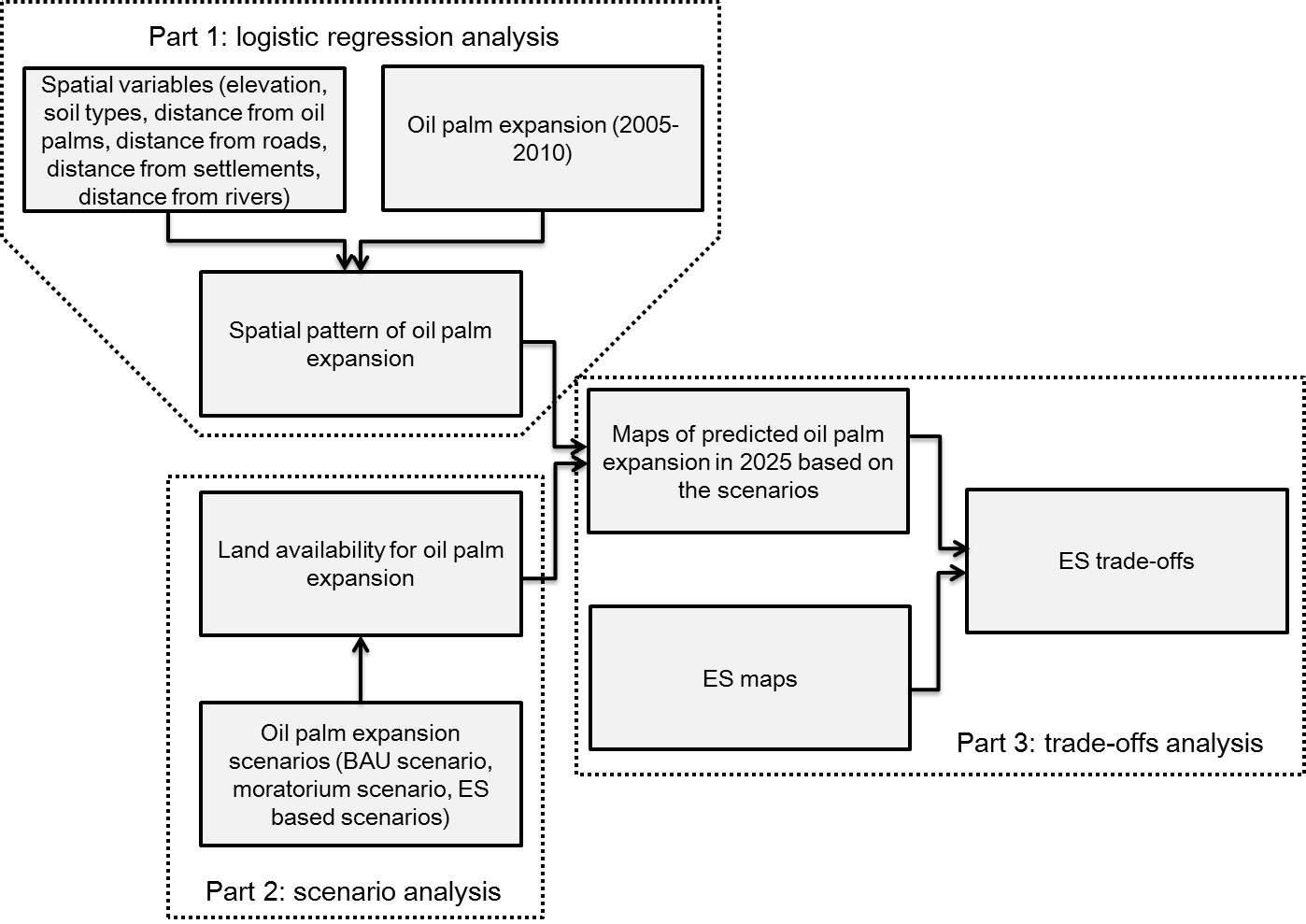


Appendix 3a. Coefficients and P values of predictors

| Predictors | Coefficients | P values |
| --- | --- | --- |
| Intercept | 2.76e+00 | < 2e16**** |
| Elevation | -1.685e-02 | < 2e-16**** |
| distance to roads | -9.477e-06 | 0.193537 |
| distance to rivers | 1.048e-04 | 4.02e-07**** |
| distance to settlements | -6.139e-05 | < 2e16**** |
| distance to existing oil palms | -3.572e-05 | < 2e16**** |
| soil type (peat) | -6.432e-01 | 0.000535**** |

Significant codes: ‘****’ 0 ‘***’ 0.001 ‘**’ 0.01 ‘*’ 0.05

Appendix 3b. The success of logistic regression model


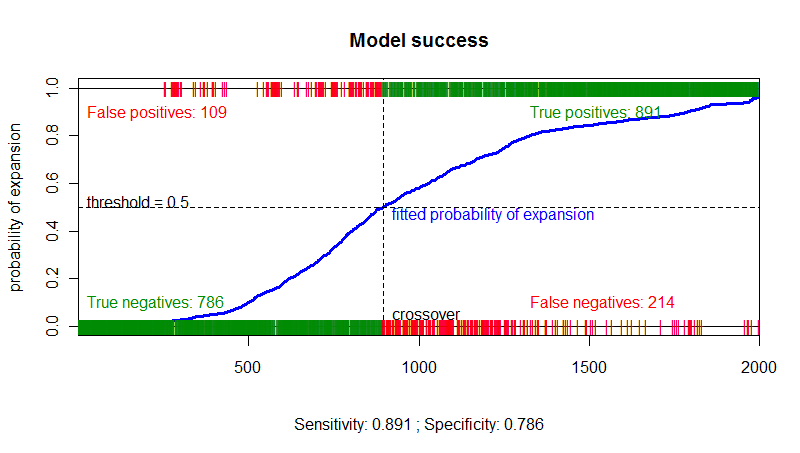


The accuracy of the model was analysed by measuring its sensitivity, specificity and AUC (Area under the ROC curve). The ROC is a graph of “sensitivity” versus (1- “specificity”) at different thresholds. The “sensitivity” denotes the ability of the model to correctly predict the presence of oil palm expansion at specific threshold, while the “specificity” does for the absence of the expansion. The maximum value of the AUC is 1 which represents a perfect accuracy. The accuracy assessment was analysed using an “R” script of Rossiter (2014).

The graph presents the success of the model with a sensitivity of 0.89 (891 out of 1000 presence points are correctly predicted) and a specificity of 0.79 (786 out of 1000 absence points are correctly predicted) at a threshold of 0.5. Overall, the AUC of the model is 0.9. This indicates the goodness of fit of the model, and the appropriateness of the model to be applied for predicting the oil palm expansion in the future.

Appendix 4. Oil palm expansion from 2000 to 2025 according to three scenarios.


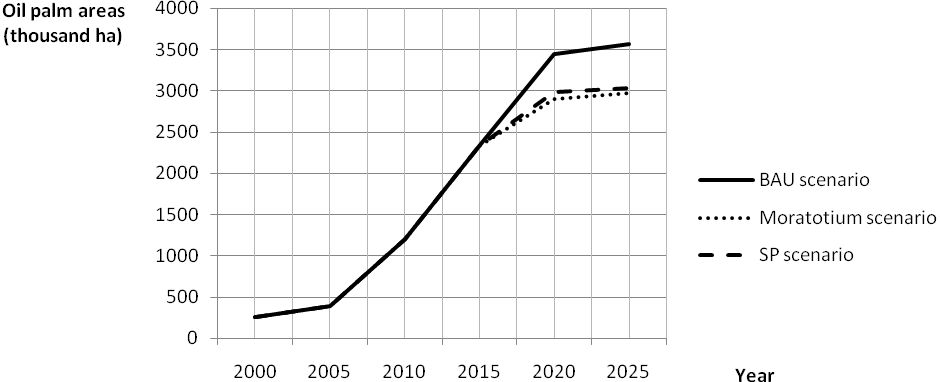


Appendix 5. Carbon balances for oil palm establishment on different land cover and soil types, + indicates sequestration, - is emission. The estimates are for the first cycle (25 years) of oil palm plantation.

|  | Land clearing (ton C/ha) | Change in soil carbon or peat decomposition (ton C/ha/year) | Fixation in oil palm plantation (ton C/ha/year) | Carbon balances (ton C/ha) | |
| --- | --- | --- | --- | --- | --- |
|  |  |  |  | for 25 years | Average per year |
| Oil palm on peat | -176.7 (forests conversion),  -11.7 (non-forests conversion)^a^ | -27.2^b^ | 1.4^a^ | -821.5 (forests conversion),  -656.5 (non-forests conversion) | -32.8 (forests conversion),  -26.3 (non-forests conversion) |
| Oil palm on mineral soil | -176.7 (forests conversion),  -11.7 (non-forests conversion)^a^ | -1.6 (forest conversion),  0.5 (non-forests conversion)^a^ | 1.4^a^ | -182.5 (forests conversion),  36.5 (non-forests conversion) | -7.3 (forests conversion), 1.5 (non-forests conversion) |

^a^ average estimates of Germer and Sauerborn (2008) / ^b^ Page et al. (2011)

Carbon balances of land conversion for oil palm depend on three aspects: land clearing, change in soil carbon, and carbon fixation by oil palm. Land clearing removes biomass (carbon) stored in former land covers (usually by burning), where conversion of forests produces higher carbon emissions. Change in soil carbon represents a balance between carbon emissions from decomposition of soil organic matter and carbon fixation from formation of soil organic matter. Oil palm establishment on peat soil, which is composed by accumulation of organic matter in a water-logged condition, requires drainage, which subsequently leads to very high carbon emissions from organic matter decomposition. The last aspect, i.e. carbon fixation by oil palm, refers to the net amount of carbon sequestered by oil palm during photosynthesis and respiration.

Appendix 6a. Value trade-offs of ecosystem services from oil palm expansion in the period 2015 – 2025 under the BAU scenario, negative value indicates reduction, values are rounded

| Types of ecosystem services | Change of area (ha) | Productivity/  quantity | Monetary value | Change of value (€/year) |
| --- | --- | --- | --- | --- |
| Oil palm on mineral soil (5 years old) | 78,200 | Productivity of 3.6 ton FFB/ha/year | Resource rent of  € -646/ ha/year (during year 0 – 4) | -50,517,200 |
| OP mineral soil (10 years old) | 614,600 | Productivity of 15.2 ton FFB/ha/year | Resource rent of  € 761/ ha/year (during year 0 – 9) | 467,710,600 |
| Oil palm on peat soil (5 years old) | 45,500 | Productivity of 3.6 ton FFB/ha/year | Resource rent of  € -924/ ha/year (during year 0 – 4) | -42,042,000 |
| Oil palm on peat soil (10 years old) | 495,600 | Productivity of 15.2 ton FFB/ha/year | Resource rent of  € 509/ ha/year (during year 0 – 9) | 252,260,400 |
| Oil palm total |  |  |  | 627,411,800 |
| Timber | -363,200 | Productivity of 0.86 m^3^/ha/year | Resource rent of  € 35/m^3^ | -10,932,320 |
| rattan | -428,000 | Productivity of 0.79 ton/ha/year | Resource rent of €104/ton | -35,164,480 |
| rice | -223,000 | Productivity of 2.2 ton/ha/year | Resource rent of  €130/ton | -63,778,000 |
| Carbon balance (conversion of forest on mineral soil) | 219,770 | Net emissions of 7.3 ton C/ha/year | Social costs of  € 88/ton C | -151,079,630 |
| Carbon balance (conversion of non-forest on mineral soil) | 440,830 | Net sequestration of 1.5 ton C/ha/year | Avoided social costs of € 88/ton C | 60,413,100 |
| Carbon balance (conversion of forest in peat soil) | 236,110 | Net emissions of 32.8 ton C/ha/year | Social costs of  € 88/ton C | -619,698,534 |
| Carbon balance (conversion of non-forest on mineral soil) | 367,650 | Net emissions of 26.3 ton C/ha/year | Social costs of  € 88/ton C | -755,253,523 |
| Carbon balance total |  |  |  | -1,465,618,590 |

The resource rent equals the revenues minus the value of intermediate consumption, and labor and the user costs of fixed assets. The user costs of fixed assets consist of consumption of fixed capital (depreciation) and the cost of capital, i.e. the opportunity cost for the money tied up in fixed assets.

The social costs of carbon (SCC), is an estimate of the monetized damages associated with the increment increase in carbon emissions in a given year (Interagency Working Group on Social Cost of Carbon, United States Government, 2013).

Appendix 6b. Value trade-offs of ecosystem services from oil palm expansion in the period 2015 – 2025 under the M scenario, negative value indicates reduction, values are rounded

| Types of ecosystem services | Change of area (ha) | Productivity/  quantity | Monetary value | Change of value (€/year) |
| --- | --- | --- | --- | --- |
| Oil palm on mineral soil (5 years old) | 76,700 | Productivity of 3.6 ton FFB/ha/year | Resource rent of  € -646/ ha/year (during year 0 – 4) | -49,548,200 |
| Oil palm on mineral soil (10 years old) | 561,100 | Productivity of 15.2 ton FFB/ha/year | Resource rent of  € 761/ ha/year (during year 0 – 9) | 426,997,100 |
| Oil palm on peat soil (5 years old) | 0 | Productivity of 3.6 ton FFB/ha/year | Resource rent of  € -924/ ha/year (during year 0 – 4) | 0 |
| Oil palm on peat soil (10 years old) | 0 | Productivity of 15.2 ton FFB/ha/year | Resource rent of  € 509/ ha/year (during year 0 – 9) | 0 |
| Oil palm total |  |  |  | 377,448,900 |
| Timber | -209,100 | Productivity of 0.86 m^3^/ha/year | Resource rent of  € 35/m^3^ | -6,293,910 |
| rattan | -390,000 | Productivity of 0.79 ton/ha/year | Resource rent of €104/ton | -32,042,400 |
| rice | -123,000 | Productivity of 2.2 ton/ha/year | Resource rent of  €130/ton | -35,178,000 |
| Carbon balance (conversion of forest on mineral soil) | 212,000 | Net emissions of 7.3 ton C/ha/year | Social costs of  € 88/ton C | -136,188,800 |
| Carbon balance (conversion of non-forest on mineral soil) | 425,800 | Net sequestration of 1.5 ton C/ha/year | Avoided social costs of € 88/ton C | 56,205,600 |
| Carbon balance (conversion of forest in peat soil) | 0 | Net emissions of 32.8 ton C/ha/year | Social costs of  € 88/ton C | 0 |
| Carbon balance (conversion of non-forest on mineral soil) | 0 | Net emissions of 26.3 ton C/ha/year | Social costs of  € 88/ton C | 0 |
| Carbon balance total |  |  |  | -79,983,200 |

Appendix 6c. Value trade-offs of ecosystem services from oil palm expansion in the period 2015 – 2025 under the SP scenario, negative value indicates reduction, values are rounded

| Types of ecosystem services | Change of area (ha) | Productivity/quantity | Monetary value | Change of value (€/year) |
| --- | --- | --- | --- | --- |
| Oil palm on mineral soil (5 years old) | 48,300 | Productivity of 3.6 ton FFB/ha/year | Resource rent of  € -646/ ha/year (during year 0 – 4) | -31,201,800 |
| Oil palm on mineral soil (10 years old) | 650,300 | Productivity of 15.2 ton FFB/ha/year | Resource rent of  € 761/ ha/year (during year 0 – 9) | 494,878,300 |
| Oil palm on peat soil (5 years old) | 0 | Productivity of 3.6 ton FFB/ha/year | Resource rent of  € -924/ ha/year (during year 0 – 4) | 0 |
| Oil palm on peat soil (10 years old) | 0 | Productivity of 15.2 ton FFB/ha/year | Resource rent of  € 509/ ha/year (during year 0 – 9) | 0 |
| Oil palm total |  |  |  | 463,676,500 |
| Timber | 0 | Productivity of 0.86 m^3^/ha/year | Resource rent of  € 35/m^3^ | 0 |
| rattan | 0 | Productivity of 0.79 ton/ha/year | Resource rent of €104/ton | 0 |
| rice | 0 | Productivity of 2.2 ton/ha/year | Resource rent of  €130/ton | 0 |
| Carbon balance (conversion of forest on mineral soil) | 0 | Net emissions of 7.3 ton C/ha/year | Social costs of  € 88/ton C | 0 |
| Carbon balance (conversion of non-forest on mineral soil) | 698,600 | Net sequestration of 1.5 ton C/ha/year | Avoided social costs of € 88/ton C | 92,215,200 |
| Carbon balance (conversion of forest in peat soil) | 0 | Net emissions of 32.8 ton C/ha/year | Social costs of  € 88/ton C | 0 |
| Carbon balance (conversion of non-forest on mineral soil) | 0 | Net emissions of 26.3 ton C/ha/year | Social costs of  € 88/ton C | 0 |
| Carbon balance total |  |  |  | 92,215,200 |
